# The Health of the Dentist

**Published:** 1869-04

**Authors:** 


### Editorial.

### "THE HEALTH OF THE DENTIST."

WE would call special attention to the article with the above caption published in this and the preceding number of the REGISTER, as one well worthy of consideration by every Dental practitioner. The writer has not descended as much into minutiæ perhaps as some would expect, or desire in such a paper; but he has so well discussed the subject in a general way, and so fully presented principles, that detailed statements would seem to be unnecessary; and still to many something in that direction would be acceptable. Every occupation in life has its difficulties, trials and involvments; these may be either physical or mental, or both. The Dentist has a liberal share of physical toil and mental labor—a large amount of hard wearing, physical labor without invigorating exercise, ordinarily so much confined as to preclude the possibility of obtaining bodily exercise of a proper kind, or of a sufficient quantity.

He who will be faithful to his trust will have much care and anxiety. The Dentist comes in more immediate contact with—by his sympathies—and is influenced by the feelings and sufferings of his patients, than is generally supposed. The Dentist of acute sensibility will often suffer as much, and in some instances, more than his patient.

That wonderful, and hitherto unexplained influence expressed according to common acceptation in likes and dislikes, exercises a wonderfully sustaining or depressing influence, and these very conditions thus brought about operate not a little to control the physical condition. Far too little attention is given to this subject. Who that has had much experience in the practice of our profession, does not realize that contact with some persons is very depressing and exhausting, while with others there is an entire absence of such exhaustion, and with some a real invigoration.

These things have their influence in respect to health. Other things, such as constraining and fatiguing position long maintained, influencing more or less the respiratory, circulatory and digestive organs, should receive some consideration. The utter impossibility of the Dentist in his operations upon the natural teeth, receiving a pure and uncontaminated atmosphere for respiration, is patent to all. His respiratory food, is largely vitiated with carbonic acid gas, with other debris thrown out of the system of his patient; this vitiation will vary exceedingly in character, in some cases being intensely offensive and exceedingly poisonous.

He who will suggest efficient means for overcoming the difficulties we have mentioned, will be a great benefactor to our profession. T.

